# Diversity of Methanogens in Animals’ Gut

**DOI:** 10.3390/microorganisms9010013

**Published:** 2020-12-23

**Authors:** Cheick Oumar Guindo, Bernard Davoust, Michel Drancourt, Ghiles Grine

**Affiliations:** 1 IHU Méditerranée Infection, 13005 Marseille, France; cheicko86@gmail.com (C.O.G.); michel.drancourt@univ-amu.fr (M.D.); 2 IRD, MEPHI, Aix-Marseille Université, 13005 Marseille, France; bernard.davoust@gmail.com; 3 Faculty of Odontology, Aix-Marseille Université, 13005 Marseille, France

**Keywords:** mammals’ digestive tract, dynamics of methanogens, sources of methanogens and zoonotic methanogens

## Abstract

Methanogens are members of anaerobe microbiota of the digestive tract of mammals, including humans. However, the sources, modes of acquisition, and dynamics of digestive tract methanogens remain poorly investigated. In this study, we aimed to expand the spectrum of animals that could be sources of methanogens for humans by exploring methanogen carriage in animals. We used real-time PCR, PCR-sequencing, and multispacer sequence typing to investigate the presence of methanogens in 407 fecal specimens collected from nine different mammalian species investigated here. While all the negative controls remained negative, we obtained by PCR-sequencing seven different species of methanogens, of which three (*Methanobrevibacter smithii*, *Methanobrevibacter millerae* and *Methanomassiliicoccus luminyensis*) are known to be part of the methanogens present in the human digestive tract. *M. smithii* was found in 24 cases, including 12/24 (50%) in pigs, 6/24 (25%) in dogs, 4/24 (16.66%) in cats, and 1/24 (4.16%) in both sheep and horses. Genotyping these 24 *M. smithii* revealed five different genotypes, all known in humans. Our results are fairly representative of the methanogen community present in the digestive tract of certain animals domesticated by humans, and other future studies must be done to try to cultivate methanogens here detected by molecular biology to better understand the dynamics of methanogens in animals and also the likely acquisition of methanogens in humans through direct contact with these animals or through consumption of the meat and/or milk of certain animals, in particular cows.

## 1. Introduction

Methanogens are archaea characterized by their unique capability in producing methane from byproducts of bacterial anaerobe fermentations, being members of anaerobe microbiota of the digestive tract microbiota of several mammals [1]. Methanogens as strict anaerobes are classified to be limited to anoxic habitats. However, some studies have shown that some methanogens are able to produce methane in soils rich in oxygen [2] and even in human microbiota [3]. Methanogens were classified into three biochemical groups based on the substrates used for hydrogen production: hydrogenotrophic, aceticlastic, and methylotrophic [2,4,5]. The group most described in human microbiota is hydrogenotrophic methanogens, which oxidize H_2,_ formate or a few simple alcohols, and reduce CO_2_ to CH_4_. 

Accordingly, methanogens gained interest in the clinical microbiology over the past years after they were detected by PCR-based methods and cultured from the gut microbiota [6,7]; their translocation in milk and urines has been further observed [8]. Moreover, methanogens have been associated with dysbiosis such as in the case of vaginosis [9], urinary tract infections [10], and anaerobe abscesses of the brain [11,12], the muscle [13], the oral cavity in the case of periodontitis, and periimplantitis [14,15] in the case of refractory sinusitis [16]. Recently, we observed blood-borne methanogens associated with endocarditis [17]. In all these situations, anaerobe bacteria were associated in the methanogen-disease process, and this observation was probably reflecting methanogen specificities, including the absolute oxygen intolerance and the necessity of bacterial fermentative products to produce methane [4,18].

Currently, 16 different methanogens have been cultured from digestive-tract microbiota of animals [19,20,21,22], and PCR-based methods of detecting species-specific sequences traced an additional 4 species [21] (Table 1).

The sources, modes of acquisition, and dynamics of digestive-tract methanogens remain poorly investigated. We previously reported that one-day newborns exhibited culturable *Methanobrevibacter smithii* (*M. smithii*) in the gastric fluid [23], suggesting a perinatal source of acquisition. Accordingly, we reported that mother milk did contain culturable *M. smithii* and culturable *Methanobrevibacter oralis* (*M. oralis*) [8]. Yet, it is unclear whether these one-day methanogens do persist along with the digestive tract of the newborns or whether this is just one of several waves of acquisition of methanogens along the first months of life [23,24,25,26,27]. Therefore, the search for methanogens sources other than mother milk is of interest.

Certain mammals (cow, sheep, donkey, horse, cat, pig, rabbit, rat, rhinoceros, baboon, monkey, and hippopotamus); birds (goose, turkey, and chicken) and insects (termites) are acknowledged to harbor digestive tract methanogens, and *M. smithii* in particular has already been detected from bovine and also from Wistar rats [21,22,28,29,30,31,32,33,34,35,36,37,38,39,40,41,42,43]. In this study, we aimed to expand the spectrum of animals that could be sources of methanogens for humans, by exploring methanogen carriage in animals.

## 2. Materials and Methods

### 2.1. Feces Samples

After the obtention of verbal consent from animals’ owners, feces samples were collected from nine different animal species, namely cat, dog, horse, sheep, rabbit, cow, pig, goat, and donkey from animals living in metropolitan France, more precisely in the Marseille metropolitan area, Southeastern France (Table 2). Dogs and cats were fed industrial dry-kibble feed; horses were fed hay + straw + pellets; sheep and goats were fed pasture (grass) and dry supplementary feed; rabbits were fed dehydrated alfalfa + hay + pellets (other vegetables, cereals, mineral salts, and vitamins); cows were fed hay + straw + pasture (grass) and whole plant maize silage; pigs were fed straw + dry pelleted feed (formula consisting mainly of maize, wheat, oats, peas, soybeans, cereals, oilseeds, and minerals), and donkeys were fed grass and hay. Feces samples were stored at +4 °C for five weeks before being processed for DNA extraction as reported below.

### 2.2. DNA Extraction and PCR Assays

DNA extraction was performed by mixing 0.2 g of each feces sample with 500 μL of G2 buffer (QIAGEN, Hilden, Germany) in an Eppendorf tube (Fisher Scientific, Illkirch, France). Then, 0.3 g of acid-washed beads ≤106 μm (Sigma-Aldrich, Saint-Quentin Fallavier, France) was added in each tube and shaken in a FastPrep BIO 101 device (MP Biomedicals, Illkirch, France) for 45 s for mechanical lysis before 10-min incubation at 100 °C. A 180 µL volume of the mixture was then incubated with 20 µL of proteinase K (QIAGEN) at 56 °C overnight before a second mechanical lysis was performed. Total DNA was finally extracted with the EZ1 Advanced XL extraction kit (QIAGEN) and 200 μL eluted volume. Sterile phosphate-buffered saline (PBS) was used as a negative control in each DNA extraction run. Extracted DNA was incorporated into real-time PCR performed using Metha_16S_2_MBF: 5′-CGAACCGGATTAGATACCCG -3′ and Metha_16S_2_MBR: 5′- CCCGCCAATTCCTTTAAGTT-3′ primers (Eurogentec, Angers, France) and a FAM_Metha_16S_2_MBP 6FAM- CCTGGGAAGTACGGTCGCAAG probe targeting the 16S DNA gene of methanogens, designed in our laboratory (Eurogentec). PCR amplification was done in a 20 μL volume including 15 μL of mix and 5 μL of extracted DNA. Five μL of ultrapure water (Fisher Scientific) was used instead of DNA in the negative controls. The amplification reaction was performed in a CFX96 thermocycler (BioRad, Marnes-la-Coquette, France) incorporating a protocol with a cycle of 50 °C for 2-min, followed by 39 cycles of 95 °C for 5-min, 95 °C for 5 s and finally 60 °C for 30 s. The PCR-sequencing was done in a 20 μL volume, including 15 μL of mix and 5 μL of extracted DNA. Five μL of ultrapure water (Fisher Scientific, Illkirch, France) was used instead of DNA in the negative controls. The amplification reaction was performed in a CFX96 thermocycler (BioRad, Marnes-la-Coquette, France) incorporating a protocol with a cycle of 50 °C for 2-min, followed by 39 cycles of 95 °C for 5-min, 95 °C for 5 s and finally 60 °C for 30 s. Amplification of the archaeal 16S rRNA gene (primers used: SDArch0333aS15, 5-TCCAGGCCCTACGGG-3 and SDArch0958aA19, 5-YCCGGCGTTGAMTCCAATT-3) was performed as previously described [8,9,33,34]. Sequencing reactions (Sangers’ method) were carried out using the BigDye Terminator, version 1.1, cycle sequencing kit DNA according to the manufacturer’s instructions (Applied Biosystems, Foster City, USA). Nucleotide sequences were assembled using Chromas Pro software, version 1.7 (Technelysium Pty Ltd., Tewantin, Australia) and compared with sequences available in the GenBank database using the online NCBI BLAST program (http://blast.ncbi.nlm.nih.gov.gate1.inist.fr/Blast.cgi). We considered the sequences as belonging to the same species if the percentage of identity was >98.7%; as different species if between 95–98.7%, and different genera if this threshold was < 95% with respect to the first hit obtained by BLAST [44].

### 2.3. Multispacer Sequence Typing

We carried out a multispacer sequence typing (MST) technique on all fecal specimens positive by PCR-sequencing as previously described in our laboratory [23,45]. PCRs were realized in a 2720 Thermal Cycler (Applied Biosystems, Foster City, California, USA) and followed all the steps described for standard PCR used for the molecular analysis of fecal specimens. Negative controls consisting of PCR mixture without DNA template were included in each PCR run. All PCR products were sequenced in both directions using the same primers as used for PCRs in a 2720 Thermal Cycler (Applied Biosystems) with an initial 1-min denaturation step at 96 °C, followed by 25 cycles denaturation for 10 s each at 96 °C, a 20 s annealing step at 50 °C, and a 4-min extension step at 60 °C. Sequencing products were purified using the MultiScreen 96-well plates Millipore (Merck, Molsheim, France), containing 5% of Sephadex G-50 (Sigma-Aldrich), and sequences were analyzed on an ABI PRISM 31309 Genetic Analyzer (Applied Biosystems, Foster City, California, USA) and edited using the ChromasPro software (version 1.42; Technelysium Pty Ltd., Tewantin, Australia). For each intergenic spacer, a spacer type (ST) was defined as a sequence exhibiting unique genetic polymorphism (SNPs and indels). MST genotypes were defined as a unique combination of the four spacer sequences [23,45].

### 2.4. Phylogenetic Analyses

Sequences were edited using ChromasPro software (ChromasPro 1.7, Technelysium Pty Ltd., Tewantin, Australia). Molecular phylogenetic and evolutionary analyses were conducted in MEGA7 as previously described [46].

### 2.5. Statistical Analyses

We used R software for data analysis (https://www.r-project.org/). The Chi 2 test was used to compare the prevalence between the different animal species with a threshold α = 0.05.

## 3. Results

In this study, a total of 407 fecal specimens collected from nine different mammalian species were investigated by real-time PCR and PCR-sequencing for the presence of methanogens using primers targeting the broad-range archaeal 16S rRNA gene.

Firstly, incorporating the 16S rRNA archaeal gene PCR primers newly designed in our laboratory into real-time PCR, we detected the presence of methanogen DNA in all animals here investigated and none of the negative controls. We found that 100.0% of cat feces specimens were positive with Ct values of 33.51 ± 1.28; 78.8% of dog feces specimens were positive with Ct values of 27.71 ± 0.94; 84.4% of horse feces specimens were positive with Ct values of 25 ± 2.95; 96.6% of sheep feces specimens were positive with Ct values of 27.19 ± 3.11; 100% of rabbit feces specimens were positive with Ct values of 27.1 ± 1.36; 100% of cow feces specimens were positive with Ct values of 24.11 ± 1.94; 100% of pig feces specimens were positive with Ct values of 22.15 ± 2.75; 80% of goat feces specimens were positive with Ct values of 19.18 ± 2.46; and 100% of donkey feces specimens were positive with Ct values of 18.82 ± 1.44 (Table 3).

Secondly, sequencing the standard PCR products was used for the precise identification of methanogens at the genus and species levels in each sample. In cats, 50/105 successfully sequenced samples yielded 20 *Methanocorpusculum aggregans* (*M. aggregans*), 13 *Methanocorpusculum labreanum (M. labreanum)*, 09 *Methanobrevibacter millerae (M. millerae)*, 04 *M. smithii*, 02 *Methanobrevibacter thaueri (M. thaueri)*, and 02 *Methanobrevibacter olleyae (M. olleyae)*. In dogs, 30/52 successfully sequenced samples yielded 13 *M. labreanum*, 06 *M. smithii*, 05 *M. aggregans*, 03 *M. thaueri*, 02 *M. millerae*, and 01 *M. olleyae*. In horses, 24/89 successfully sequenced samples yielded 11 *M. aggregans*, 10 *M. olleyae*, 01 *M. smithii*, 01 *M. millerae*, and 01 *M. labreanum*. In sheep, 28/29 successfully sequenced samples yielded 23 *M. labreanum*, 03 *M. millerae*, 01 *M. smithii*, and 01 *M. aggregans*. In rabbits, 2/2 successfully sequenced samples yielded 02 *M. thaueri*. In cows, 44/57 successfully sequenced samples yielded 22 *M. aggregans*, 11 *M. millerae*, 06 *M. labreanum,* and 05 *M. thaueri*. In pigs, 25/64 successfully sequenced samples yielded 12 *M. smithii*, 09 *M. millerae*, 03 *Methanomassiliicoccus luminiyensis* (*M. luminiyensis),* and 01 *M. olleyae*. In goats, 4/5 successfully sequenced samples yielded 03 *M. labreanum* and 01 *M. aggregans*. Finally, in donkeys, 4/4 successfully sequenced samples yielded 04 *M. aggregans* (Table 4).

**Figure 1:**
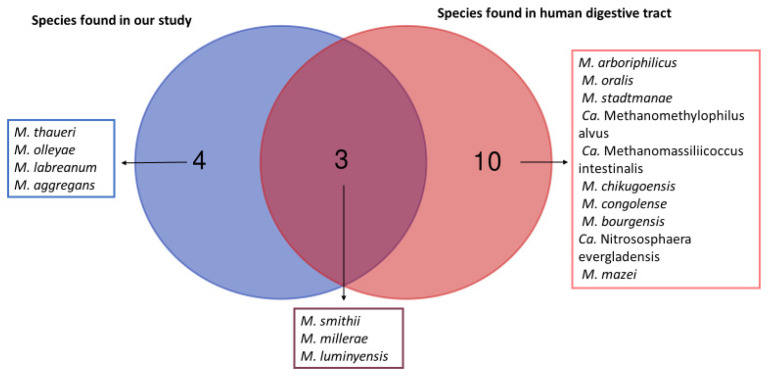
Venn diagram between the methanogens found in our study and those known from the human digestive tract. This Venn diagram shows which species of methanogens are found in common in humans and in animal samples analyzed in this study and which species of methanogens are found exclusively in animals and exclusively in humans.

We obtained a total of seven different species of methanogens in our study as illustrated with Venn diagrams (Figure 1).). The Venn diagram shows which species of methanogens are found in common in humans and in animal samples analyzed in this study, and which species of methanogens are found exclusively in animals and exclusively in humans. Indeed, three methanogens species (*M. smithii, M. millerae,* and *M. luminyensis*) are known to be part of the methanogens present in the human digestive tract. The remaining four (*M. thaueri*, *M. olleyae, M. labreanum* and *M. aggregans*) are not known to date in humans. However, we did not find in our study the other 10 species of methanogens present in the human digestive tract, including *Methanobrevibacter arboriphilicus*, *M. oralis*, *Methanosphaera stadtmanae* (*M. stadtmanae)*, *Candidatus* Methanomethylophilus alvus (*Ca.* Methanomethylophilus alvus), *Candidatus* Methanomassiliicoccus intestinalis (*Ca*. Methanomassiliicoccus intestinalis), *Methanoculleus chikugoensis* (*M. chikugoensis*), *Methanobacterium congolense* (*M. congolense*), *Methanoculleus bourgensis* (*M. bourgensis*), *Candidatus* Nitrososphaera evergladensis (*Ca.* Nitrososphaera evergladensis), and *Methanosarcinia mazei* (*M. mazei*).

**Figure 2:**
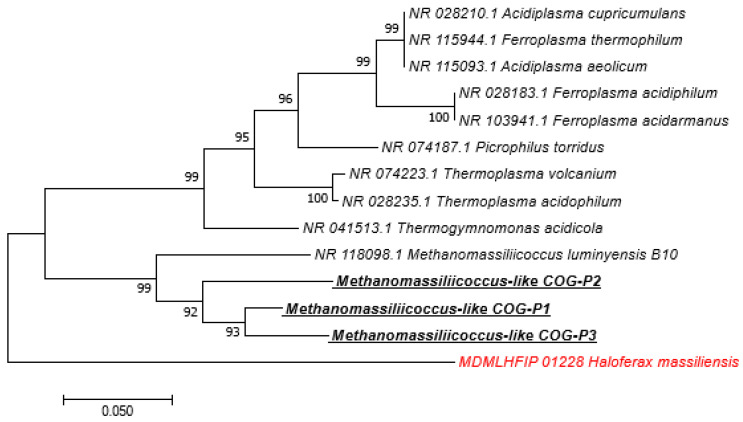
Molecular phylogenetic analysis, based on 16S rRNA partial gene, showed the position of *Methanomassiliicoccus-like* sequences detected in feces of pigs. The evolutionary history was inferred using the Neighbor-Joining method. The optimal tree with the sum of branch length = 0.82722721 is shown. The percentage of replicate trees in which the associated taxa clustered together in the bootstrap test (1.000 replicates) are shown next to the branches. The tree is drawn to scale, with branch lengths in the same units as those of the evolutionary distances used to infer the phylogenetic tree. The evolutionary distances were computed using the Maximum Composite Likelihood method and are in the units of the number of base substitutions per site. The analysis involved 14 nucleotide sequences. All positions containing gaps and missing data were eliminated. There were a total of 415 positions in the final dataset. Evolutionary analyses were conducted in MEGA7. Bootstrap values ≥ 95% are indicated at nodes. In red: out of group. Species highlighted: methanogens species detected in this study.

Among the 211 sequences obtained, 153 (72.51%) of them have an identity percentage greater than 99%, 43 (20.37%) have an identity percentage lower than 98.7%, and 15 (7.10%) have an identity percentage lower than 95% (Table 5). The phylogenetic trees of sequences obtained with a percentage identity lower than 98.7% and sequences with a percentage identity lower than 95% indicated new species and new genera, respectively (Figure 2 and Supplementary Figures). We obtained 24 *M. smithii* by PCR-sequencing including 12/24 (50%) in pigs, 6/24 (25%) in dogs, 4/24 (16.66%) in cats, and 1/24 (4.16%) in both sheep and horses. Genotyping the 24 *M. smithii* revealed five different genotypes. Genotype 1 was found in 8/24 (33.33%); genotype 2 in 10/24 (41.66%); genotype 3 in 4/24 (16.66%); and genotypes 4 and 5 in 1/24 (4.16%) each (Table 6).

## 4. Discussion

It is known and published that methanogens colonize the gastrointestinal tract of certain mammals, particularly herbivorous ones [35]. Most methanogens identified in mammals belong to the phylum Euryarchaeota, with a high percentage of the species *M. smithii* [36], a species being the most prevalent one in humans [47]. Our report is the largest one showing the presence of methanogens in nine mammals in the same study. Our results confirmed the published data on the presence of methanogens in the digestive tract of cats, dogs, horses, cows, sheep, rabbits, goats, pigs, and donkeys [21,22,28,29,30,31,32,33,34,36,37,38]. In addition, all methanogens found in this study belong to the phylum Euryarchaeota, which is in accordance with the results obtained in studies conducted on the human digestive tract [6,48]. Our results give an insight on the concentration of methanogens present in the intestinal microbiota of each animal species analyzed and on the prevalence of methanogens in domestic animals by humans.

The results of the analysis of the 16S RNA sequences obtained from our samples show that there is a real diversity of methanogenic archaea genera (*Methanosphaera*, *Methanocorpusculum*, *Methanocalculus*, *Methanoculleus*, *Methanogenium*, *Methanoplanus*, *Methanolacinia*, *Methanobacterium*, *Methanomicrobium*, *Methanomassiliicoccus* and *Methanobrevibacter*) in the digestive tract of animals (cats, dogs, horses, sheep, cows, rabbits, goats, pigs, and donkeys) as in humans [6,48]. All sequences with a percentage lower than 98.7% have been deposited in the GenBank database (accession no MT587812 to MT587864) and EBI database (accession no MT793590; MT819603; MT822292; MT822293; and MT822482).

*Methanomassiliicoccus luminiyensis* was known to colonize the human digestive tract, and it has never been detected in animals’ digestive tracts [46]. For the first time, this study demonstrated the presence of the species *M. luminiyensis* in pigs and not in the other animals investigated here. These results could be explained by the fact that the pig is an omnivore, which means that its diet is close to that of humans compared to other animals. In addition, 50% of *M. smithii* in our study was found in pigs, indicating that *M. smithii* was the most prevalent methanogen in the digestive tract of pigs, consistent with work carried out in humans where the high prevalence of *M. smithii* in the digestive tract has been demonstrated [45,49].

These results are representative of the methanogen community present in the digestive tract of certain animals domesticated by humans, and other future studies must be done to try to cultivate methanogens detected here by molecular biology to better understand the dynamics of methanogens in animals. The possible ways of methanogens’ acquisition in humans could be contact with animals and/or through consumption of milk/dairy products of certain animals, in particular cows, since a recent study demonstrated an association between the acquisition of *M. smithii* in children and the consumption of dairy products [50].

## Figures and Tables

**Table 1:** Methanogens found in digestive tract microbiota of animals.

Methanogens Species Obtained by Culture	Additional Species Detected by PCR-Based Methods But Not by Culture
*Methanosarcina* sp. *Methanobacterium formicicum* *Methanomicrobium mobile* *Methanosarcina barkeri* *Methanobacterium bryantii* *Methanobrevibacter, ruminantium* *Methanobrevibacter millerae* *Methanobrevibacter olleyae* *Methanoculleus olentangyi* *Methanobrevibacter woesei* *Methanobrevibacter gottschalkii* *Methanobrevibacter thaueri* *Methanobrevibacter wolinii* *Methanobrevibacter cuticularis* *Methanobrevibacter curvatus* *Methanobrevibacter filiformi*	*Methanobrevibacter smithii* *Methanimicrococcus* spp. *Methanosphaera* spp. *Methanobacterium* spp.

**Table 2:** Details of 407 feces samples here investigated for the presence of methanogens.

Origin of Samples	Species	Collected Number per Sample	Collection Sites
**Cat**	*Felis silvestris catus*	105	Marseille
**Dog**	*Canis lupus*	52	Marseille
**Horse**	*Equus caballus*	89	Marseille and Carnoux
**Sheep**	*Ovis aries*	29	Bourganeuf
**Rabbit**	*Oryctolagus cuniculus*	2	Allauch
**Cow**	*Bos taurus*	57	Bourganeuf and Allauch
**Pig**	*Sus scrofa domesticus*	64	Avignon
**Goat**	*Capra aegagrus hircus*	5	Allauch
**Donkey**	*Equus asinus*	4	Allauch

**Table 3:** Comparison of prevalence based on real-time PCR between animal species.

Animal Species	Number of Samples Analyzed	Number of Positive Samples by RT-PCR	Prevalence [IC 95%]	*p*-Value
**Cat**	105	105	100.0 [96.5–100.0]	9.9 × 10^–8^
**Dog**	52	41	78.8 [65.3–88.9]	
**Horse**	89	75	84.4 [75.0–91.1]	
**Sheep**	29	28	96.6 [82.2–99.9]	
**Rabbit**	2	2	100.0 [15.8–100.0]	
**Cow**	57	57	100.0 [93.7–100.0]	
**Pig**	64	64	100.0 [94.4–100.0]	
**Goat**	5	4	80.0 [28.4–99.5]	
**Donkey**	4	4	100.0 [39.8–100.0]	

**Table 4:** Comparison of prevalence based on PCR-sequencing between animal species.

Animal Species	Number of Samples Analyzed	Number of Positive Samples by PCR-Sequencing	Prevalence [IC 95%]	*p*-Value
**Cat**	105	50	47.6 [37.8–57.6]	1.4 × 10^–12^
**Dog**	52	30	57.7 [43.2–71.3]	
**Horse**	89	24	27.0 [ 18.1–37.4]	
**Sheep**	29	28	96.6 [82.2–99.9]	
**Rabbit**	2	2	100.0 [15.8–100.0]	
**Cow**	57	44	77.2 [64.2–87.3]	
**Pig**	64	25	39.1 [27.1–52.1]	
**Goat**	5	4	80.0 [28.4–99.5]	
**Donkey**	4	4	100.0 [39.8–100.0]	

**Table 5:** Percentage of identity among the sequences obtained.

Animal Species	Percentage > 99%	Percentage < 98.7%	Percentage < 95%
**Cat**	50	0	0
**Dog**	30	0	0
**Horse**	7	15	2
**Sheep**	18	7	3
**Rabbit**	2	0	0
**Cow**	29	12	3
**Pig**	16	4	5
**Goat**	0	2	2
**Donkey**	1	3	0

**Table 6:** Summary of the results of multispacer sequence typing.

				Genotypes			
Samples	Origin	Collection Sites	Spacer 1	Spacer 2	Spacer 3	Spacer 4	Spacer Type *
1	Sheep	Bouganeuf			×		1
2	Horse	Marseille			×		1
3	Pig	Avignon	×	×	×	×	2
4	Pig	Avignon	×	×	×	×	2
5	Pig	Avignon	×	×	×	×	2
6	Pig	Avignon	×	×	×	×	2
7	Pig	Avignon	×	×	×		3
8	Pig	Avignon	×	×			4
9	Pig	Avignon		×	×		5
10	Pig	Avignon			×		1
11	Pig	Avignon	×	×	×	×	2
12	Pig	Avignon	×	×	×		3
13	Pig	Avignon	×	×	×	×	2
14	Pig	Avignon	×	×	×	×	2
15	Dog	Marseille	×	×	×	×	2
16	Dog	Marseille	×	×	×	×	2
17	Dog	Marseille	×	×	×		3
18	Dog	Marseille	×	×	×		3
19	Dog	Marseille	×	×	×	×	2
20	Dog	Marseille			×		1
21	Cat	Marseille			×		1
22	Cat	Marseille			×		1
23	Cat	Marseille			×		1
24	Cat	Marseille			×		1

Genotyping these *M. smithii* strains revealed the presence of five different genotypes. * Spacer type was determined according to reference [23,45].

## Supplementary Materials

The following are available online at https://www.mdpi.com/2076-2607/9/1/13/s1.
