# Supplementary material for: Diversity of Methanogens in Animals’ Gut
Source: Microorganisms. 2020 Dec 23;9(1):13. doi: 10.3390/microorganisms9010013 (PMC7822204; doi:10.3390/microorganisms9010013)
Supplement: Supplementary file 1 [file microorganisms-09-00013-s001.pdf]

Supplementary Figures

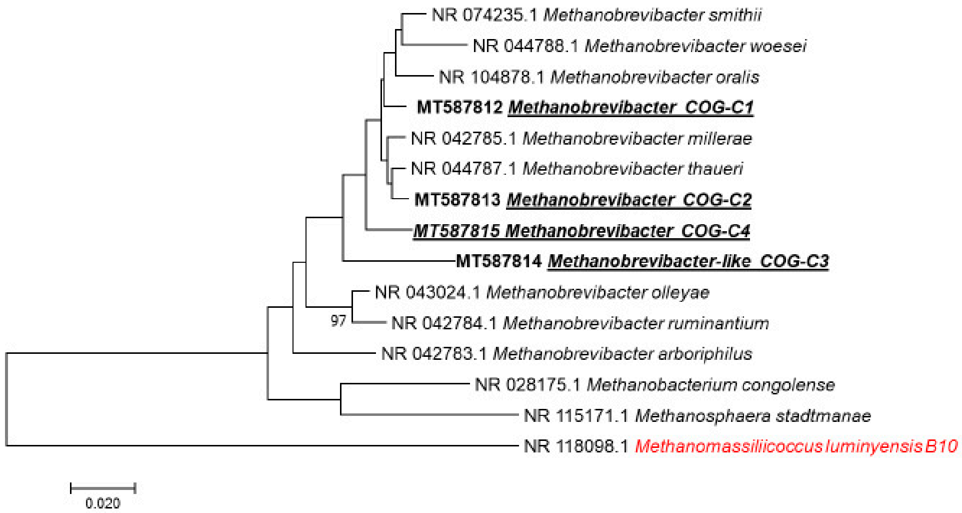

Supplementary Figure 1. Molecular phylogenetic analysis, based on 16S rRNA partial gene, showed the position of *Methanobrevibacter* sequences detected in feces of cow. The evolutionary history was inferred using the Neighbor-Joining method. The optimal tree with the sum of branch length = 0.56972409 is shown. The percentage of replicate trees in which the associated taxa clustered together in the bootstrap test (1.000 replicates) are shown next to the branches. The tree is drawn to scale, with branch lengths in the same units as those of the evolutionary distances used to infer the phylogenetic tree. The evolutionary distances were computed using the Maximum Composite Likelihood method and are in the units of the number of base substitutions per site. The analysis involved 15 nucleotide sequences. All positions containing gaps and missing data were eliminated. There was a total of 447 positions in the final dataset. Evolutionary analyses were conducted in MEGA7. Bootstrap values ≥ 95% are indicated at nodes. In red: out of group. Species highlighted: methanogens species detected in this study.

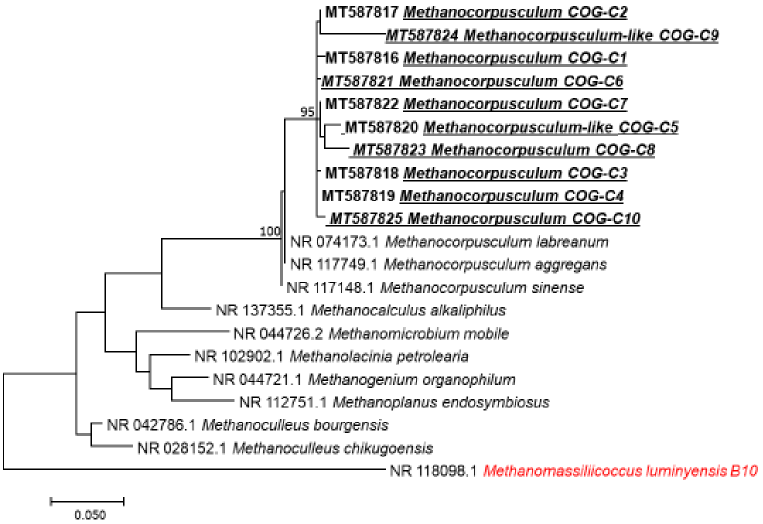

Supplementary Figure 2. Molecular phylogenetic analysis, based on 16S rRNA partial gene, showed the position of *Methanocorpusculum* sequences detected in feces of cow. The evolutionary history was inferred by using the Maximum Likelihood method based on the Kimura 2-parameter model. The tree with the highest log likelihood (−1869.71) is shown. The percentage of trees in which the associated taxa clustered together is shown next to the branches. Initial tree(s) for the heuristic search were obtained automatically by applying Neighbor-Join and BioNJ algorithms to a matrix of pairwise distances estimated using the Maximum Composite Likelihood (MCL) approach, and then selecting the topology with superior log likelihood value. The tree is drawn to scale, with branch lengths measured in the number of substitutions per site. The analysis involved 21 nucleotide sequences. All positions containing gaps and missing data were eliminated. There was a total of 375 positions in the final dataset. Evolutionary analyses were conducted in MEGA7. Bootstrap values ≥ 95% are indicated at nodes. In red: out of group. <u>Species highlighted:</u> methanogens species detected in this study.

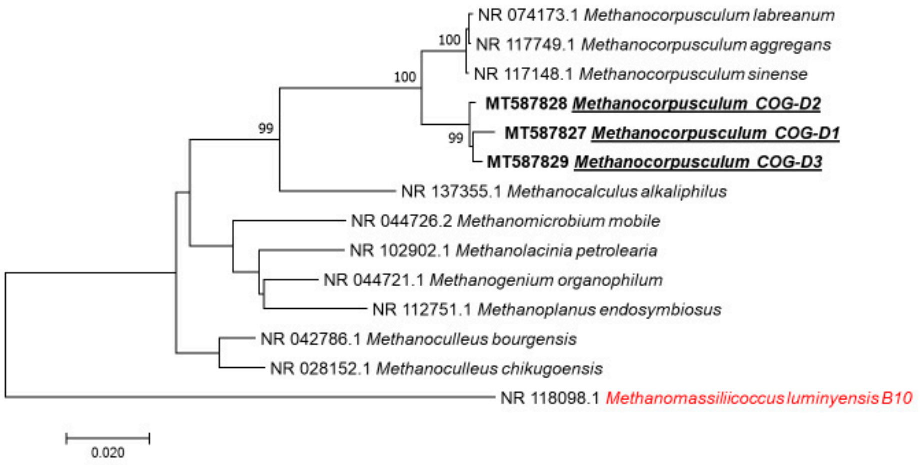

Supplementary Figure 3. Molecular phylogenetic analysis, based on 16S rRNA partial gene, showed the position of *Methanocorpusculum* sequences detected in feces of donkey. The evolutionary history was inferred using the Neighbor-Joining method. The optimal tree with the sum of branch length = 0.41522849 is shown. The percentage of replicate trees in which the associated taxa clustered together in the bootstrap test (1.000 replicates) are shown next to the branches. The tree is drawn to scale, with branch lengths in the same units as those of the evolutionary distances used to infer the phylogenetic tree. The evolutionary distances were computed using the Maximum Composite Likelihood method and are in the units of the number of base substitutions per site. The analysis involved 14 nucleotide sequences. All positions containing gaps and missing data were eliminated. There was a total of 476 positions in the final dataset. Evolutionary analyses were conducted in MEGA7. Bootstrap values ≥ 95% are indicated at nodes. In red: out of group. Species highlighted: methanogens species detected in this study.

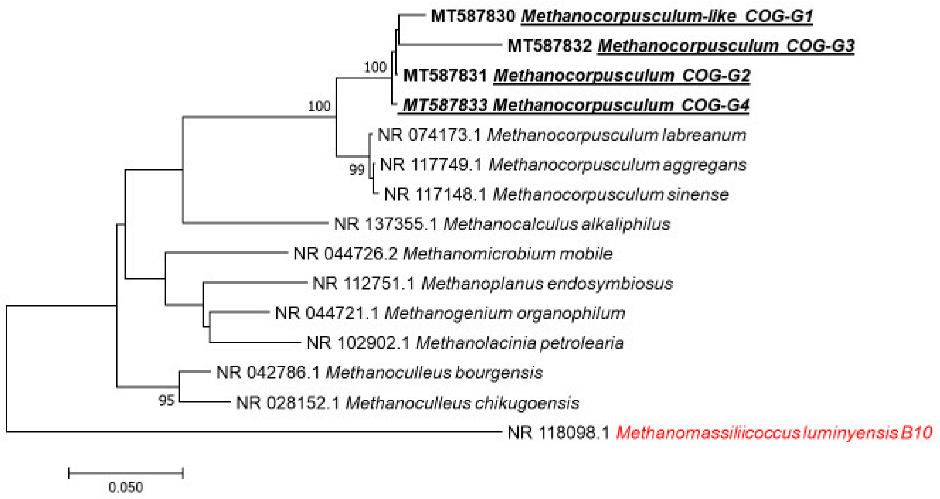

Supplementary Figure 4. Molecular phylogenetic analysis, based on 16S rRNA partial gene, showed the position of *Methanocorpusculum* sequences detected in feces of goat. The evolutionary history was inferred using the Neighbor-Joining method. The optimal tree with the sum of branch length = 0.79665377 is shown. The percentage of replicate trees in which the associated taxa clustered together in the bootstrap test (1.000 replicates) are shown next to the branches. The tree is drawn to scale, with branch lengths in the same units as those of the evolutionary distances used to infer the phylogenetic tree. The evolutionary distances were computed using the Maximum Composite Likelihood method and are in the units of the number of base substitutions per site. The analysis involved 15 nucleotide sequences. All positions containing gaps and missing data were eliminated. There was a total of 427 positions in the final dataset. Evolutionary analyses were conducted in MEGA7. Bootstrap values ≥ 95% are indicated at nodes. In red: out of group. Species highlighted: methanogens species detected in this study.

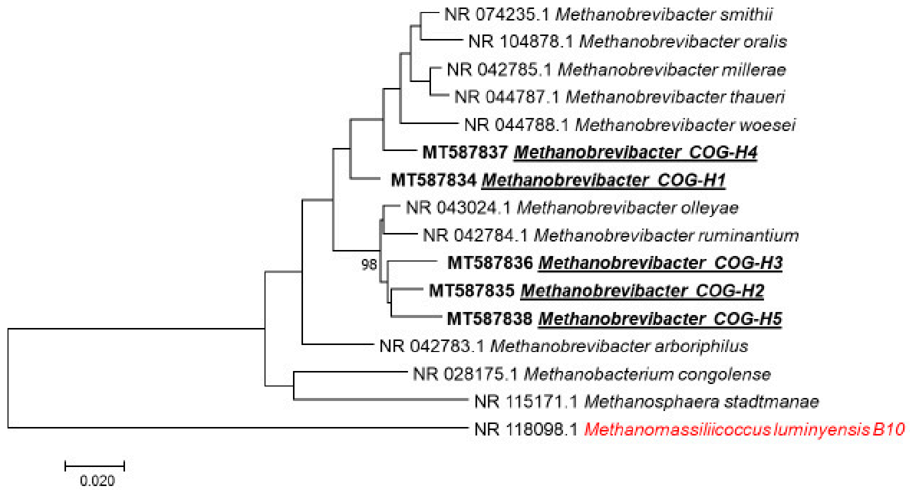

Supplementary Figure 5. Molecular phylogenetic analysis, based on 16S rRNA partial gene, showed the position of *Methanobrevibacter* sequences detected in feces of horse. The evolutionary history was inferred using the Neighbor-Joining method. The optimal tree with the sum of branch length = 0.58360989 is shown. The percentage of replicate trees in which the associated taxa clustered together in the bootstrap test (1.000 replicates) are shown next to the branches. The tree is drawn to scale, with branch lengths in the same units as those of the evolutionary distances used to infer the phylogenetic tree. The evolutionary distances were computed using the Maximum Composite Likelihood method and are in the units of the number of base substitutions per site. The analysis involved 16 nucleotide sequences. All positions containing gaps and missing data were eliminated. There was a total of 408 positions in the final dataset. Evolutionary analyses were conducted in MEGA7. Bootstrap values ≥ 95% are indicated at nodes. In red: out of group. Species highlighted: methanogens species detected in this study.

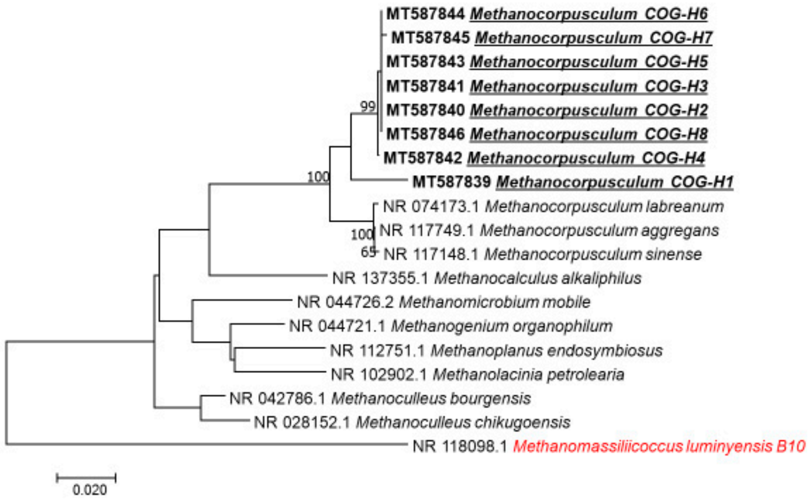

Supplementary Figure 6. Molecular phylogenetic analysis, based on 16S rRNA partial gene, showed the position of *Methanocorpusculum* sequences detected in feces of horse. The evolutionary history was inferred using the Neighbor-Joining method. The optimal tree with the sum of branch length = 0.52514892 is shown. The percentage of replicate trees in which the associated taxa clustered together in the bootstrap test (1.000 replicates) are shown next to the branches. The tree is drawn to scale, with branch lengths in the same units as those of the evolutionary distances used to infer the phylogenetic tree. The evolutionary distances were computed using the Maximum Composite Likelihood method and are in the units of the number of base substitutions per site. The analysis involved 19 nucleotide sequences. All positions containing gaps and missing data were eliminated. There was a total of 417 positions in the final dataset. Evolutionary analyses were conducted in MEGA7. Bootstrap values ≥ 95% are indicated at nodes. In red: out of group. Species highlighted: methanogens species detected in this study.

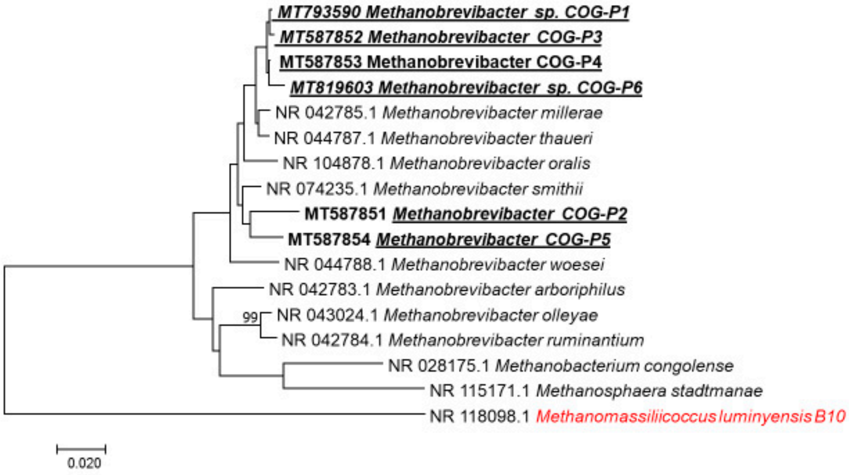

Supplementary Figure 7. Molecular phylogenetic analysis, based on 16S rRNA partial gene, showed the position of *Methanobrevibacter* sequences detected in feces of pig. The evolutionary history was inferred using the Neighbor-Joining method. The optimal tree with the sum of branch length = 0.56411513 is shown. The percentage of replicate trees in which the associated taxa clustered together in the bootstrap test (1.000 replicates) are shown next to the branches. The tree is drawn to scale, with branch lengths in the same units as those of the evolutionary distances used to infer the phylogenetic tree. The evolutionary distances were computed using the Maximum Composite Likelihood method and are in the units of the number of base substitutions per site. The analysis involved 17 nucleotide sequences. All positions containing gaps and missing data were eliminated. There was a total of 467 positions in the final dataset. Evolutionary analyses were conducted in MEGA7. Bootstrap values ≥ 95% are indicated at nodes. In red: out of group. Species highlighted: methanogens species detected in this study.

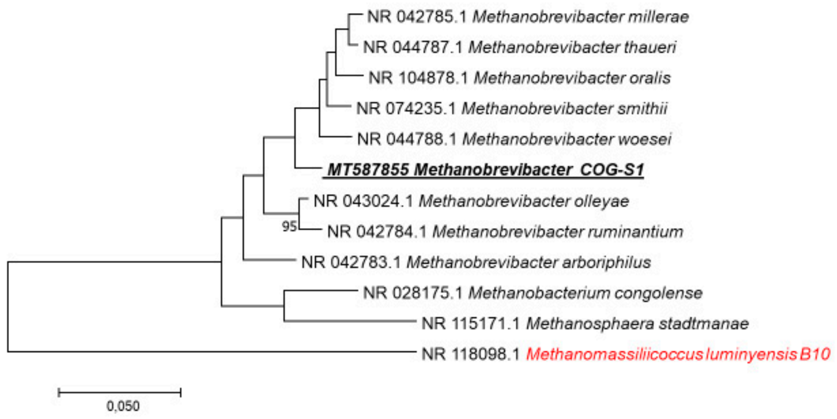

Supplementary Figure 8. Molecular phylogenetic analysis, based on 16S rRNA partial gene, showed the position of *Methanobrevibacter* sequence detected in feces of sheep. The evolutionary history was inferred by using the Maximum Likelihood method based on the Kimura 2-parameter model. The tree with the highest log likelihood (-1972.37) is shown. The percentage of trees in which the associated taxa clustered together is shown next to the branches. Initial tree(s) for the heuristic search were obtained automatically by applying Neighbor-Join and BioNJ algorithms to a matrix of pairwise distances estimated using the Maximum Composite Likelihood (MCL) approach, and then selecting the topology with superior log likelihood value. The tree is drawn to scale, with branch lengths measured in the number of substitutions per site. The analysis involved 12 nucleotide sequences. All positions containing gaps and missing data were eliminated. There was a total of 554 positions in the final dataset. Evolutionary analyses were conducted in MEGA7. Bootstrap values ≥ 95% are indicated at nodes. In red: out of group. Species highlighted: methanogens species detected in this study.

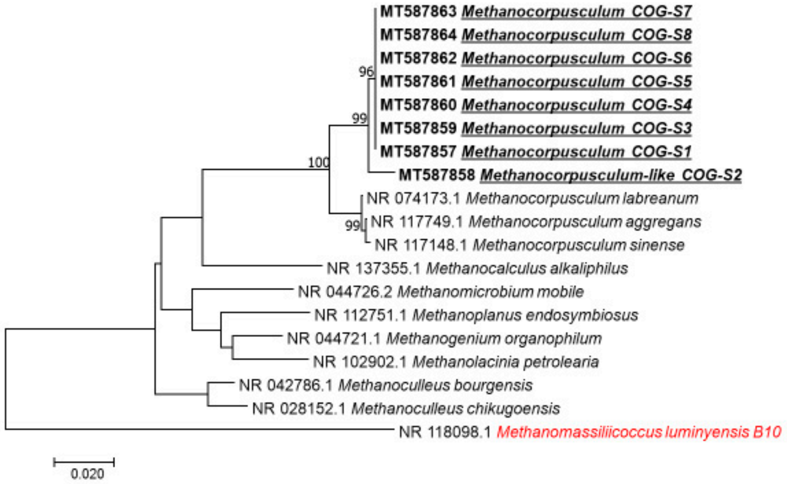

Supplementary Figure 9. Molecular phylogenetic analysis, based on 16S rRNA partial gene, showed the position of *Methanocorpusculum* sequences detected in feces of sheep. The evolutionary history was inferred using the Neighbor-Joining method. The optimal tree with the sum of branch length = 0.48477167 is shown. The percentage of replicate trees in which the associated taxa clustered together in the bootstrap test (1.000 replicates) are shown next to the branches. The tree is drawn to scale, with branch lengths in the same units as those of the evolutionary distances used to infer the phylogenetic tree. The evolutionary distances were computed using the Maximum Composite Likelihood method and are in the units of the number of base substitutions per site. The analysis involved 19 nucleotide sequences. All positions containing gaps and missing data were eliminated. There was a total of 441 positions in the final dataset. Evolutionary analyses were conducted in MEGA7. Bootstrap values ≥ 95% are indicated at nodes. In red: out of group. Species highlighted: methanogens species detected in this study.
